# Associations between abdominal obesity and the risk of stroke in Chinese older patients with obstructive sleep apnea: Is there an obesity paradox?

**DOI:** 10.3389/fnagi.2022.957396

**Published:** 2022-09-12

**Authors:** Xiaofeng Su, Kailiang Li, Ling Yang, Yang Yang, Yinghui Gao, Yan Gao, JingJing Guo, Junling Lin, Kaibing Chen, Jiming Han, Lin Liu

**Affiliations:** ^1^Department of Pulmonary and Critical Care Medicine of the Second Medical Center and National Clinical Research Center for Geriatric Diseases, Chinese PLA General Hospital, Beijing, China; ^2^Sichuan College of Traditional Chinese Medicine, Mianyang, China; ^3^Medical College, Yan’an University, Yan’an, China; ^4^Cardiology Department of the Second Medical Center and National Clinical Research Center for Geriatric Diseases, Chinese PLA General Hospital, Beijing, China; ^5^PKU-UPenn Sleep Center, Peking University International Hospital, Beijing, China; ^6^Department of General Practice, 960th Hospital of PLA, Jinan, China; ^7^Sleep Medicine Center, Department of Respiratory and Critical Care Medicine, Peking University People’s Hospital, Beijing, China; ^8^Department of Respiratory and Critical Care Medicine, Beijing Chaoyang Hospital, Capital Medical University, Beijing, China; ^9^Sleep Center, The Affiliated Hospital of Gansu University of Chinese Medicine, Lanzhou, China

**Keywords:** abdominal obesity, obstructive sleep apnea, elder, stroke, prospective study

## Abstract

**Background and purpose:**

Abdominal obesity (AO) is a well-known independent risk factor for stroke in the general population although it remains unclear in the case of the elderly, especially in Chinese older patients with obstructive sleep apnea (OSA), considering the obesity paradox. This study aimed to investigate the association between AO and stroke among Chinese older patients with OSA.

**Methods:**

Data were collected from January 2015 to October 2017, and 1,290 older patients (age 60–96 years) with OSA (apnea–hypopnea index ≥ 5 events/h on polysomnography) were consecutively enrolled from sleep centers at six hospitals, evaluated for AO defined as waist circumference (WC) using the standardized criteria for the Chinese population, and followed up prospectively for a median period of 42 months. Logistic regression and Cox regression analyses were used to determine the cross-sectional and longitudinal associations between AO and stroke risk in these participants and different groups of the severity of OSA.

**Results:**

Participants with AO had a higher prevalence of stroke at baseline. A higher incidence of stroke during a median follow-up period of 42 months in participants with AO than in participants without AO (12.4% *vs*. 6.8% and 8.3% *vs.* 2.4%, respectively; both *P* < 0.05) was predicted. Cross-sectional analysis revealed an association between AO and stroke (odds ratio [OR]1.96, 95% confidence interval [CI] 1.31–2.91), which was stronger among participants with moderate OSA only (OR 2.16, 95%CI 1.05–4.43). Cox regression analysis showed that, compared to participants without AO, participants with AO had a higher cumulative incidence of stroke (hazard ratio [HR] 2.16, 95% CI 1.12–4.04) during a median follow-up of 42 months, and this association was observed in patients with severe OSA only (HR 3.67, 95% CI 1.41–9.87) but not for individuals with mild OSA (HR = 1.84, 95% CI 0.43–6.23) and moderate OSA (HR = 1.98, 95% CI 0.73–6.45).

**Conclusion:**

The risk of stroke is associated with AO among Chinese older patients who have OSA, both at baseline and during follow-up, and the strength of the association varied by OSA severity. Active surveillance for early detection of AO could facilitate the implementation of stroke-preventive interventions in the Chinese older OSA population.

## Introduction

Obstructive sleep apnea (OSA) is the most common sleep disorder and is a leading health concern due to its strong association with the increasing burden of major adverse cardiovascular and cerebrovascular diseases and all-cause mortality (Chan et al., 2019; Gottlieb and Punjabi, 2020). Furthermore, OSA confers a higher risk of stroke-predisposing conditions, including vascular aging, transient ischemic attack, acute ischemic stroke, and wake-up stroke, for which the current OSA treatment strategies remain inadequate (Koo et al., 2016; Bravata et al., 2018; Catalan-Serra et al., 2019; Lisan et al., 2021). The public health burden of these complications remains largely because of their higher prevalence and incidence in patients with OSA, especially in the older population. Thus, a better understanding of the risk factors of OSA is essential for developing effective preventive strategies against stroke in older patients with OSA.

Abdominal obesity (AO) is common in patients with OSA, and AO increases the risk of OSA. The results from two observational longitudinal studies confirmed a strong association between AO and OSA, whereas another study reported a stronger, more significant association in younger than in older individuals (Nakagawa et al., 2011; Unal et al., 2019; Zhao et al., 2019; Balat et al., 2020; Liu et al., 2021; Qin et al., 2021). Moreover, the health risks of patients with AO differ from that of patients with non-AO. First, individuals with AO have a higher risk for metabolic syndrome, which is associated with cerebrovascular diseases, such as stroke (Gaudio et al., 2017; Park et al., 2018). Second, AO is potentially related to both brain structure and function (Després et al., 2008). The relationship between AO and stroke risk has been investigated in several studies, all of which were conducted in the general population or non-OSA populations (Suk et al., 2003; Abete et al., 2015; Rodríguez-Campello et al., 2017; Liu S. et al., 2020; Zhai et al., 2020). Suk et al. (2003) showed that, compared with the body mass index (BMI), AO was a stronger risk factor for stroke and increased the overall odds ratio (OR) of stroke by threefold. Zhai et al. (2020) reported that waist circumference (WC), an AO index measure, was an important predictor of all-cause mortality, including stroke-related mortality, independent of the BMI. Rodríguez-Campello et al. (2017) found that AO was associated with stroke risk in only women, which indicated a sex-specific stroke risk of AO in the general population, and this finding is aligned with the results of the Spanish EPIC cohort study (Abete et al., 2015). Notably, Liu S. et al. (2020) demonstrated significant associations between obesity, regardless of general adiposity or AO and the risk of incident stroke in Chinese participants during a median follow-up of 12 years. Although the abovementioned studies examined a direct association between AO and stroke, studies that simultaneously evaluated the association between AO and the prevalence risk and/or incidence of stroke in patients with OSA, especially in the older population, are limited. AO is associated with a higher risk of stroke, whereas this is presenting a controversy in older populations. Numerous studies in free-living older populations and older individuals with chronic disease, including type 2 diabetes, coronary heart disease (CHD), heart failure, and cancer, reported that obese individuals have a better prognosis than non-obese individuals, a phenomenon termed the “obesity paradox” (Gregg et al., 2004; Lavie et al., 2009). The suggested explanations for this survival advantage are increased metabolic reserve, which can meet the metabolic needs of the disease, and lipoproteins, such as cholesterol, that can bind and remove endotoxins and neutralize inflammation (Bailly et al., 2020).

Given that the majority of the patients with OSA have AO, this obesity paradox may contribute to stroke outcomes and should be considered an important confounding factor. Thus, the present study was conducted with an aim to assess the association between AO and the prevalence of stroke among Chinese older patients with OSA at baseline and to determine the long-term effect of AO on incident stroke in a cohort of older OSA patients without stroke at baseline.

## Materials and methods

### Study design and participants

The project is a Chinese population-based, multicenter, prospective, observational study to assess the association of AO with stroke in older patients with OSA aged ≥ 60 years. The study began consecutively recruiting subjects on 1 January 2015 from sleep centers of six hospitals in China using a clinic’s electronic classification system, and the data were cutoff on 30 October 2017. This study collected baseline information including basic demographics, clinical medical characteristics, and sleep parameters. OSA was defined as an apnea-hypopnea index (AHI) ≥ 5 events/h.

**FIGURE 1:**
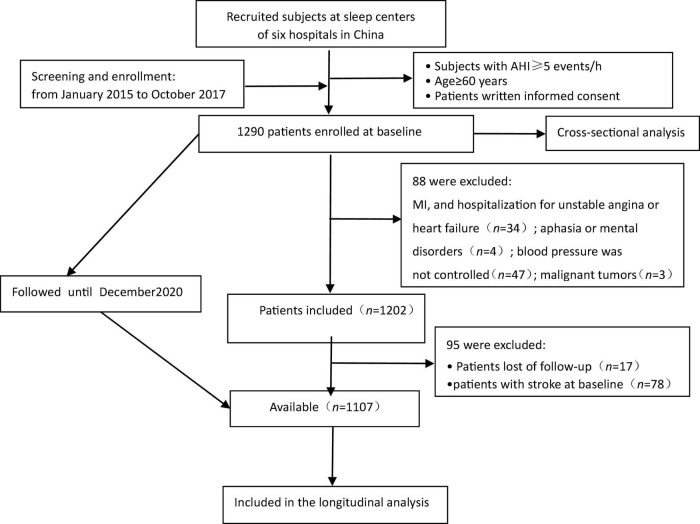
Study flowchart. AHI, the apnea-hypopnea index; MI, myocardial infarction.

For the current study, a total of 1,290 patients with OSA were enrolled in six hospitals in six areas of China, including the General Hospital of the Chinese People’s Liberation Army (PLA, Haidian District, Beijing, *n* = 313), the Peking University International Hospital (*n* = 238), and the Peking University People’s Hospital (Changping District, Beijing, *n* = 242), the Beijing Chaoyang Hospital affiliated to Capital Medical University (Chaoyang District, Beijing, China, *n* = 337), the 960th Hospital of PLA (JiNan, Shandong Province, *n* = 48), and the affiliated Hospital of Gansu University of Chinese Medicine (Lanzhou, Gansu Province, *n* = 112), who had completed polysomnography (PSG) study within 7 days after admission, at baseline, during which we performed a cross-sectional analysis. After following up prospectively for approximately 4 years, we excluded 88 patients based on the following criteria for the longitudinal analysis: (1) previous history of myocardial infarction and hospitalization for unstable angina or heart failure (*n* = 34); (2) patients with malignant tumors (*n* = 3); (3) patients with aphasia mental disorders (*n* = 4); and those whose blood pressure was not controlled with antihypertensive agents (*n* = 47). Furthermore, we excluded 95 patients who were lost during the follow-up (*n* = 17) and patients with stroke at baseline (*n* = 78). Eventually, the final sample included in the prospective analysis contained 1,107 participants, and the study participant-selection flowchart is presented in Figure 1. The cohort was followed up from the OSA diagnosis of PSG assessment to December 2020.

This study is reported following the Strengthening the Reporting of Observational Studies in Epidemiology guideline, was conducted in compliance with the tenets underlying the Declaration of Helsinki, and was approved by the Ethics Committee of Chinese PLA General Hospital (S2019-352-01); all participants provided written informed consent for study participation.

### Overnight sleep study

Polysomnography is a gold standard for diagnosis of OSA and all participants completed standardized full nocturnal PSG testing (from 21:00 to 07:00 the next day) in a sleep laboratory within 7 days after admission (Liu et al., 2022). Participants were asked to strictly abstain from consuming caffeine, sedatives, and hypnotic drugs in the 24 h preceding the full-night sleep study. The sleep analysis was performed on-site using laboratory-based PSG equipment (Compumedics, Melbourne, VIC, Australia) and standard methods that have been described previously (Liu et al., 2022). All essential parameters of the sleep tests with specific respiration events were recorded and included electroencephalography, electrooculography, electrocardiography, nasal and oral airflow, thoracic/abdominal movements, pulse oxygen saturation, tracheal microphone for snoring, body position, and sleep parameters, such as the AHI, oxygen desaturation indices (ODI), mean oxygen saturation (MSpO_2_), lowest oxygen saturation (LSpO_2_), and total sleep time (TST). Apnea was defined as a complete cessation of respiratory airflow for more than 10 s, whereas hypopnea was defined as at least a 30% reduction of air flow accompanied by a 4% or greater decrease in SaO_2_ or arousal. The AHI was defined as the number of episodes of apnea and hypopnea per hour of sleep. Oxygen desaturation index (ODI) was defined as the average number of arterial oxygen saturation dips ≥ 4%/h. TST was defined as the time spent in all sleep stages. LSpO_2_ was defined as the lowest value of whole oxygen saturation observed during sleep. MSpO_2_ was defined as the mean value of whole oxygen saturation observed during sleep. TSA90 was defined as the total sleep time spent at oxygen saturation levels below 90% (Shaikh et al., 2013). Sleep scores were calculated in accordance with the Guideline of the American Academy of Sleep Medicine (Berry et al., 2012). The categories of OSA severity were defined using conventional clinical cutoffs of the AHI, which is the gold standard for OSA diagnosis: AHI < 5, 5–15, 15–30, and >30 events/h as no, mild, moderate, and severe OSA, respectively (Berry et al., 2012; Patil et al., 2019).

### Covariates

Demographics included age, sex, BMI, waist–hip ratio, neck circumference, and WC. The waist circumference was measured according to the WHO recommended method (Who, 1995). The subject stood with feet apart, using a non-elastic soft ruler, along the midpoint of the line connecting the lower edge of the twelfth rib and the upper edge of the iliac crest in the mid-axillary line, wrapped horizontally for 1 week, and was measured at the end of calm expiration, with an accurate reading of 0.1 cm. The height reading was accurate to 0.1 cm and the weight reading was accurate to 0.1 kg. When measuring the hip circumference, both legs were held together and upright, both arms were naturally lowered, and the tape measure was placed horizontally in front of the pubic symphysis and behind the most convex part of the gluteus maximus. It was measured by a tape measure around the widest part of the hip for 1 week, and the result was accurate at 0.1 cm. Waist-to-hip ratio (WHR) was calculated as WC divided by hip circumference. All physical examination measurers received uniform training.

Lifestyle variables included self-reported current smoking (consecutively or cumulatively smoking for > 6 months in the past year) and alcohol consumption (drinking ≥ 50 g/week of alcohol for ≥ 6 months).

Comorbidities included CHD, diabetes, hypertension, hyperlipidemia, atrial fibrillation (AF), carotid atherosclerosis (CAS), chronic obstructive pulmonary disease (COPD), and hyperlipidemia. Hypertension was defined as a previous diagnosis of hypertension or the use of antihypertensive medication (Ma et al., 2021). Hyperlipidemia was defined as total cholesterol ≥ 220 mg/dl, TG ≥ 150 mg/dl, or undergoing treatment for hyperlipidemia using the Chinese Guidelines for the management (Joint committee issued Chinese guideline for the management of dyslipidemia in adults, 2016). Atrial fibrillation was determined by self-reporting or atrial fibrillation on ECG at the inpatient center based on the ESC guidelines (Kirchhof et al., 2016). CHD and COPD were determined by a record of a relevant diagnostic clinical (Read) code indicating the presence of the condition (Charlson et al., 1987). Carotid atherosclerosis (CAS) was determined based on the carotid intima-media thickness (CIMT) measured by color Doppler ultrasound diagnostic instrument: when CIMT ≥ 1 mm was determined as carotid atherosclerotic plaque formation (Chambless et al., 2000). Diabetes had been diagnosed by a physician based on the guidelines provided by the Diagnosis and Classification of Diabetes Mellitus of American Diabetes Association (No authors listed, 1997).

All participants underwent comprehensive clinical assessment at baseline, and the clinical data that were collected were screened, assessed, and extracted using predesigned electronic case report forms by at least two medical professionals who were blinded to the patients’ PSG results. The categories of covariates are listed in Supplementary Table S1.

### Assessment of abdominal obesity

Abdominal obesity was defined as WC ≥ 90 cm and ≥85 cm in men and women, respectively, according to the standardized criteria for Chinese individuals (Zhou, 2002). The study cohort in the prospective analysis was subdivided into two groups: (i) a group with AO and (ii) a group without AO, all of whom have completed the median follow-up of 42 months (range 1–72 months).

### Follow-up and stroke assessment

All participants were followed-up prospectively for approximately 4 years after the full baseline assessment. Patients or their proxies were contacted initially at 1-, 3-, 6-, and 12-month intervals, and every 6 months thereafter (at least 3 months and up to 1 year) until December 2020 or death *via* telephonic follow-up, a clinic visit, or medical chart review, which was performed independently by at least two investigators who were blinded to the patients’ clinical condition, PSG results, and study group.

The predefined endpoint in our study was stroke. Strokes were recorded both at baseline and the median follow-up of 42-month. Older participants who reported two or more stroke events in multiple follow-up notes were uniformly counted as having one relevant event, and we extracted data from the longest follow-up period after the endpoint event. Stroke was defined as both ischemic and hemorrhagic stroke using the Diagnostic Criteria of Cerebrovascular Diseases in China (2019) (Chinese Society of Neurology, and Chinese Stroke Society, 2019; (Su et al., 2021). The prevalence of stroke was defined as the percentage of older patients with OSA who had stroke at baseline. The incidence of stroke was estimated as the percentage of patients who were newly diagnosed with stroke during the follow-up period, excluding patients who reported a history of stroke at baseline. Stroke was preliminarily identified based on the self*-*reported medical history (shared by patients or their proxies). Next, during a clinic visit or by medical chart review, we obtained the participant’s CT or MRI report to verify the diagnosis of stroke. Study events of all participants were adjudicated by the clinical event committee.

### Statistical analysis

Continuous variables at baseline are presented as mean ± SD or median (interquartile range 25th and 75th percentiles) in our study, and intergroup differences at baseline were compared using the Mann–Whitney *U* or the chi-square test. Logistic regression analysis was performed to obtain the ORs and 95% confidence intervals (CIs) between AO and stroke risk in total participants and different OSA (mild, moderate, and severe) groups that were stratified according to the AHI. Three models were created to examine the relationship between AO and stroke: Model 1, which was unadjusted; Model 2, adjusted for age and sex; and Model 3, further adjusted for BMI, neck circumference, drinking status, smoking status, WHR, WC, BMI, sleep parameters, and baseline self-reported comorbidities. We used three Cox regression models to examine the association between AO and hazard ratios (HRs) of stroke during a median follow-up of 42 months in the whole cohort as well as in different OSA groups. AO was considered both a continuous and categorical variable during the analysis of all models.

Additionally, all older patients with hypertension received antihypertensive drugs according to Chinese guidelines for the management of hypertension during follow-up (Sun et al., 2017). In this study, patients with hypertension were divided into two categories: treated but with uncontrolled hypertension and hypertension controlled with medication. Forty-seven patients whose blood pressure was not controlled with antihypertensive agents were excluded, whereas we did not exclude patients who had hypertension but whose blood pressure was controlled in the normal range. In a systematic review of stroke risk factors, a history of hypertension or uncontrolled hypertension conferred an increase in stroke risk, but clearly, well-controlled hypertension has a lower risk of stroke compared with uncontrolled hypertension (Gorenek et al., 2017). Three models were created to examine the relationship between AO and stroke: Model 1, which was unadjusted; Model 2, adjusted for age and sex; and we adjusted for as many variables related to stroke as possible in Model 3, adjusted for BMI, neck circumference, drinking status, smoking status, waist–hip ratio, WC, BMI, sleep parameters of MSpO_2_, LSpO_2_, TST, TSA90, ODI, AHI, and baseline self-reported comorbidities of CHD, COPD, diabetes, carotid atherosclerosis, atrial fibrillation, and hypertension. All *p*-values were two-tailed, and statistical significance was set at 0.05. SPSS (version 25.0, SPSS Inc., Chicago, IL, United States) was used for all analyses.

## Results

### Cross-sectional analysis of the association between abdominal obesity and stroke

**FIGURE 2:**
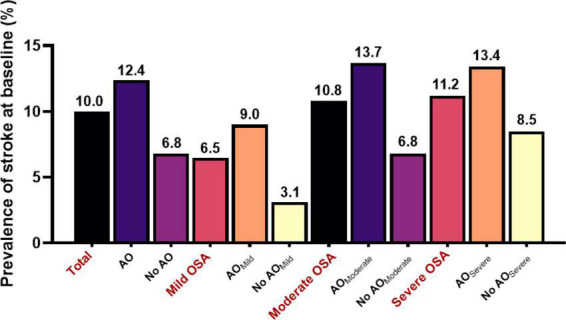
Prevalence of stroke at baseline among older patients with OSA.

All 1, 290 participants (age, median 66 [range 66–96] years) who were included in the cross-sectional analysis underwent successful overnight PSG; at baseline, 23.7, 30.1, and 46.2% of the participants had mild, moderate, and severe OSA, respectively. The prevalence of stroke was 6.5, 10.8, and 11.2% for mild, moderate, and severe OSA, respectively, and was 10.0% for all participants. The prevalence of stroke in OSA patients with AO tended to increase compared with those without AO (12.4% *vs*. 6.8%; *P* < 0.05), and this trend was consistent across different OSA groups and became stronger among participants with moderate OSA (*P* < 0.05; Figure 2).

Table 1 presents the baseline characteristics by OSA severity-based groups in participants with and without stroke. Older OSA patients with AO had a higher waist–hip ratio, BMI, and WC in each OSA group (all *P* < 0.05). A statistically significant difference was observed between stroke and non-stroke older participants in the specific OSA subgroup as follows: older OSA patients with stroke had a higher median neck circumference than those without stroke (only in the moderate OSA group); the ratios of AO (in the mild and moderate OSA groups) and the proportion of comorbidities of hypertension (both in the moderate and in the severe OSA groups), CHD (only in the mild OSA group), hyperlipidemia (in the severe OSA group), AF (only in the severe OSA group), and COPD (only in the moderate OSA group) were significantly higher between older participants with stroke compared to those without stroke, whereas no statistically significant difference was noted between the stroke and non-stroke groups in specific OSA groups for all other selected characteristics (*P* > 0.05).

**TABLE 1:** Comparisons of participants’ characteristics according to the severity of OSA groups and stroke at baseline (*n* = 1,290).

	Mild OSA (*n* = 306)			Moderate OSA (*n* = 388)			Severe OSA (*n* = 596)		
			
	Stroke (*n* = 20)	No stroke (*n* = 286)	*P*-value	Stroke (*n* = 42)	No stroke (*n* = 346)	*P*-value	Stroke (*n* = 67)	No stroke (*n* = 529)	*P*-value
Age, y	65.5 (60.5,71)	67.0 (64.0,72.0)	0.152	69.0 (65.0,77.0)	65.0 (62.0,72.0)	0.641	66.0 (64.0,76.0)	65.0 (62.0,69.0)	0.218
Men, %	9 (45.0)	160 (55.9)	0.341	33 (78.6)	200 (57.8)	0.009	52 (77.6)	344 (65.0)	0.040
BMI, kg/m2	27.8 (26.3,29.5)	25.0 (22.6,27.4)	0.003	28.4 (25.66,29.8)	25.5 (23.5,27.7)	0.001	29.7 (26.5,33.8)	27.5 (25.3,30.3)	0.034
NC, mm	39.3 (36.0,43.8)	39.0 (36.0,43.0)	0.397	41.0 (35.0,44.0)	40.0 (37.0,44.0)	0.042	43.0 (38.5,44.0)	42.0 (38.0,45.0)	0.878
WC, mm	99.0 (92.3,117.5)	90.0 (80.0,98.0)	0.001	98.0 (86.0,105.3)	90.0 (78.4,99.0)	0.006	98.0 (82.0,110.0)	90.0 (80.0,99.0)	0.001
Waist-hip ratio, %	1.0 (0.8,1.0)	0.9 (0.8,1.0)	0.015	1.0 (0.8,1.1)	0.9 (0.8,1.0)	0.011	0.9 (0.8,1.1)	0.8 (0.8,1.0)	0.014
Drinking, *n* (%)	2 (10.0)	28 (9.8)	0.976	4 (9.5)	39 (11.3)	0.733	17 (25.4)	68 (12.9)	0.006
Smoking, *n* (%)	4 (20.0)	55 (19.2)	0.933	8 (19.0)	73 (21.1)	0.757	14 (20.9)	139 (26.3)	0.342
AHI, events/h	9.7 (7.7,12.0)	9.3 (6.7,11.9)	0.516	21.2 (18.8,25.9)	21.8 (18.1,30.0)	0.978	47.6 (38.1,56.8)	48.8 (37.3,61.6)	0.984
TSA90, min	9.6 (1.8,178.0)	3.4 (0.8,30.1)	0.567	16.3 (2.6,67.2)	9.4 (2.3,26.83)	0.741	42.8 (14.6,87.1)	34.0 (9.7,116.0)	0.805
TST, h	7.3 (6.8,7.4)	7.1 (6.2,7.4)	0.387	7.2 (5.9,7.9)	7.1 (6.3,7.7)	0.609	7.4 (6.3,8.3)	7.2 (6.1,7.8)	0.472
ODI, events/h	10.6 (7.9,15.3)	8.0 (4.6,11.3)	0.314	21.0 (13.1,33.0)	17.3 (12.4,23.0)	0.104	42.0 (33.0,56.4)	41.5 (30.0,55.1)	0.054
MSpO_2_,%	92.0 (89.0,95.0)	94.0 (92.0,95.0)	0.879	94.0 (90.5,95.0)	94.0 (92.0,95.0)	0.321	93.0 (90.0,94.0)	93.0 (91.0,94.0)	0.617
LSpO_2_, %	84.0 (74.0,87.0)	84.0 (80.0,87.0)	0.209	80.0 (73.5,86.0)	82.0 (76.5,85.0)	0.798	75.0 (65.0,80.5)	75.0 (67.0,81.0)	0.199
AO, *n* (%)	16 (80.0)	162 (56.6)	0.041	31 (73.8)	196 (56.6)	0.033	45 (67.2)	292 (55.2)	0.063
Hypertension, *n* (%)	15 (75.0)	173 (60.5)	0.197	31 (73.8)	299 (57.5)	0.042	51 (76.1)	336 (63.5)	0.042
CHD, *n* (%)	0 (0.0)	55 (19.2)	0.030	13 (31.0)	84 (24.3)	0.345	20 (29.9)	132 (25.0)	0.386
Hyperlipidemia, *n* (%)	6 (30.0)	90 (31.5)	0.891	11 (26.2)	92 (26.6)	0.956	25 (37.3)	135 (25.5)	0.040
AF, *n* (%)	1 (5.0)	20 (7.0)	0.910	6 (14.3)	28 (8.1)	0.176	6 (9.0)	62 (11.7)	0.012
CAS, *n* (%)	9 (45.0)	87 (30.4)	0.174	16 (38.1)	85 (24.6)	0.059	17 (25.4)	118 (22.3)	0.572
Diabetes, *n* (%)	3 (15.0)	57 (19.9)	0.591	94 (31.0)	81 (23.4)	0.281	16 (23.9)	149 (28.2)	0.515
COPD, *n* (%)	1 (5.0)	24 (8.4)	0.592	9 (21.4)	23 (6.6)	0.003	6 (9.0)	30 (5.7)	0.288

Associations between AO and stroke that were detected in the cross-sectional analysis are shown in Table 2. AO was associated with increased odds of stroke among older patients with OSA in the crude model (OR 2.04, 95% CI 1.36–3.06), the partially adjusted model (OR 2.02, 95% CI 1.35–3.02), and the fully adjusted model (OR 1.96, 95% CI 1.31–32.91). Furthermore, WC as AO judgment criteria with an increased risk of stroke in the overall participants, and the unadjusted, partially adjusted, and fully adjusted ORs (95% CI) were 1.03 (1.02–1.05), 1.03 (1.02–1.04), and 1.02 (1.01–1.04), respectively, per 1 SD increase in WC (Table 3).

**TABLE 2:** Logistic regression model for stroke according to AO among older patients with OSA at baseline (*n* = 1,290).

	Model 1		Model 2		Model 3	
			
	*OR* (95*%CI*)	*P*-value	*OR* (95*%CI*)	*P*-value	*OR* (95*%CI*)	*P*-value
**Total participants**						
OR per 1 mm	1.03 (1.02, 1.05)	<0.001	1.03 (1.02, 1.04)	0.001	1.02 (1.01, 1.04)	0.011
Abdominal obesity	2.04 (1.36, 3.06)	0.001	2.02 (1.35, 3.02)	0.015	1.96 (1.31, 2.91)	0.011
**Mild OSA**						
OR per 1 mm	1.03 (1.01,1.05)	0.006	1.03 (1.02,1.05)	0.001	1.02 (1.01,1.04)	0.010
Abdominal obesity	3.06 (0.99, 9.38)	0.050	2.94 (0.86, 9.05)	0.052	2.89 (0.74,8.96)	0.063
**Moderate OSA**						
OR per 1 mm	1.03 (1.01, 1.05)	0.004	1.03 (1.01, 1.05)	0.005	1.03 (1.01, 1.05)	0.021
Abdominal obesity	2.45 (1.09, 4.65)	0.029	2.28 (1.10, 4.70)	0.027	2.16 (1.05, 4.43)	0.036
**Severe OSA**						
OR per 1 mm	1.03 (1.02, 1.05)	0.001	1.03 (1.02, 1.04)	0.010	1.02 (1.01, 1.04)	0.013
Abdominal obesity	1.66 (0.99, 2.83)	0.051	1.64 (0.90, 2.55)	0.054	1.44 (0.70, 1.95)	0.062

OR, odds ratio; CI, confidence interval.

Model 1 was unadjusted.

Model 2 adjusted for age and gender.

Model 3 adjusted for BMI, neck circumference, drinking status, smoking status, waist-hip ratio, waist circumference, BMI, sleep parameters, and baseline self-reported chronic diseases.

**TABLE 3:** Comparisons of participants’ characteristics according to the severity of OSA groups and AO during follow-up (*n* = 1,107).

	Mild OSA (*n* = 305)			Moderate OSA (*n* = 370)			Severe OSA (*n* = 432)		
			
	AO (*n* = 177)	No AO (*n* = 128)	*P*-value	AO (*n* = 215)	No AO (*n* = 155)	*P*-value	AO (*n* = 255)	No AO (*n* = 177)	*P*-value
Age, y	66.0 (63.0,71.0)	67.0 (64.0,72.8)	0.066	65.0 (61.0,71.0)	66.0 (62.0,73.0)	0.098	66.0 (62.0,71.0)	64.0 (61.0,67.0)	0.002
Men, %	88 (49.4)	81 (63.3)	0.016	112 (52.1)	108 (69.7)	0.001	158 (62.2)	127 (71.8)	0.039
BMI, kg/m2	25.6 (23.4,27.5)	24.4 (22.0,27.5)	0.016	25.7 (23.8,18.1)	25.5 (23.4,27.7)	0.454	27.6 (25.4,30.1)	27.6 (25.4,31.5)	0.347
NC, mm	39.0 (36.0,43.0)	38.0 (35.0,42.9)	0.252	40.0 (37.0,45.0)	40.0 (37.0,44.0)	0.727	42.0 (37.4,44.0)	43.0 (38.0,44.0)	0.096
WC, mm	98.0 (92.0,104.0)	78.0 (70.0,83.8)	<0.001	99.0 (93.0,109.0)	78.0 (70.0,80.0)	<0.001	99.0 (92.0,106.0)	79.0 (68.5,81.0)	<0.001
Waist-hip ratio, %	1.0 (0.9,1.1)	0.8 (0.7,0.9)	<0.001	1.0 (0.9,1.1)	0.7 (0.7,0.8)	<0.001	1.0 (0.9,1.1)	0.8 (0.7,0.8)	<0.001
Drinking, *n* (%)	11 (6.2)	19 (14.8)	0.012	15 (7.0)	23 (14.8)	0.014	22 (8.7)	38 (21.5)	0.004
Smoking, *n* (%)	31 (17.4)	28 (21.9)	0.329	41 (19.1)	33 (21.3)	0.598	63 (24.8)	57 (32.2)	0.092
AHI, events/h	9.4 (6.9,11.7)	8.9 (6.6,12.3)	0.690	21.7 (17.9,25.4)	21.8 (18.7,26.5)	0.151	49.5 (39.2,64.1)	45.6 (35.3,58.1)	0.007
TSA90, min	3.0 (0.7,19.9)	4.8 (0.9,66.7)	0.084	8.8 (2.1,26.4)	10.3 (2.6,33.0)	0.264	35.6 (10.6,128.7)	34.0 (10.3,92.7)	0.678
TST, h	7.0 (6.2,7.4)	7.2 (6.5,7.4)	0.119	7.0 (6.6,7.5)	7.2 (6.5,7.9)	0.037	7.2 (6.1,7.8)	7.3 (6.2,8.0)	0.123
ODI, events/h	7.8 (4.3,10.6)	9.0 (5.9,13.7)	0.002	17.0 (12.0,23.5)	18.3 (13.2,23.2)	0.415	42.5 (30.4,57.0)	39.7 (30.0,52.2)	0.238
MSpO_2_, %	94.0 (93.0,96.0)	93.0 (91.0,95.0)	0.002	94.0 (92.0,95.0)	94.0 (92.0,95.0)	0.303	92.5 (90.0,94.0)	93.0 (91.0,95.0)	0.181
LSpO_2_, %	84.5 (81.0,87.0)	84.0 (79.0,87.0)	0.572	81.0 (76.0,85.0)	81.0 (76.0,85.0)	0.983	74.0 (66.0,81.0)	79.0 (71.0,82.0)	0.601
Hypertension, *n* (%)	109 (61.2)	79 (61.7)	0.932	131 (60.9)	89 (57.4)	0.497	167 (65.7)	100 (56.5)	0.052
CHD, *n* (%)	30 (16.9)	25 (19.5)	0.547	51 (23.7)	38 (24.5)	0.860	45 (17.7)	46 (26.0)	0.038
Hyperlipidemia, *n* (%)	54 (30.3)	42 (32.8)	0.645	56 (26.0)	40 (25.8)	0.959	64 (25.2)	39 (22.0)	0.449
AF, *n* (%)	7 (4.0)	15 (11.7)	0.012	18 (8.4)	14 (9.0)	0.118	18 (7.1)	24 (13.6)	0.039
CAS, *n* (%)	42 (23.6)	54 (42.2)	0.001	50 (23.3)	44 (28.4)	0.263	42 (16.5)	22 (12.4)	0.238
Diabetes, *n* (%)	35 (19.7)	25 (19.5)	0.977	47 (21.9)	39 (25.2)	0.458	71 (28.0)	35 (19.8)	0.019
COPD, *n* (%)	9 (5.1)	15 (12.5)	0.019	13 (6.0)	17 (11.0)	0.087	14 (5.5)	3 (21.7)	0.045

BMI, body mass index; NC, neck circumference; WC, waist circumference; WHR, waist/hip ratio; AHI, the apnea-hypopnea index; ODI, the oxygen desaturation index; MSpO_2_, the mean pulse oxygen saturation; LSpO_2_, the lowest pulse oxygen saturation; TST, total sleep time; TSA90, the duration of time with SaO_2_ < 90%; AO, abdominal obesity; CHD, coronary heart disease; AF, atrial fibrillation; CAS, carotid atherosclerosis; COPD, chronic obstructive pulmonary disease.

We observed that these associations between AO and stroke varied with the severity of OSA. A onefold increase in WC was associated with a 2, 3, and 2% increase in the odds of stroke (*P* < 0.05) after adjusting for age, sex, BMI, neck circumference, drinking status, smoking status, waist–hip ratio, WC, BMI, sleep parameters, and baseline self-reported comorbidities in the mild, moderate, and severe OSA groups. The OR of AO for stroke increased significantly (OR 2.16, 95% CI 1.05–4.43) for the moderate OSA group in the fully adjusted model, although there were no significant associations between AO and odds of stroke in the mild and severe OSA groups.

### Abdominal obesity increased the risk of stroke in longitudinal analysis

**FIGURE 3:**
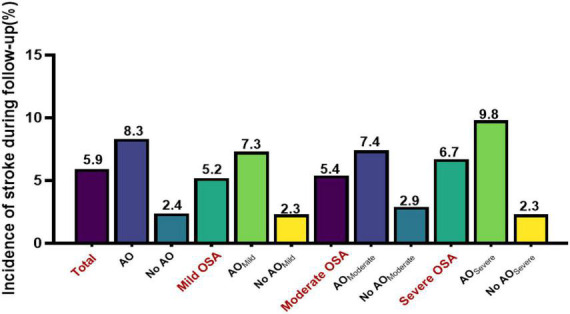
Cumulative incidence of stroke during follow-up among older patients with OSA.

After the exclusion of patients according to predefined criteria, 34 patients were excluded because of a history of myocardial infarction and hospitalization for unstable angina or heart failure; three patients with malignant tumors were excluded; four patients with aphasia mental disorders were excluded; 47 patients were excluded because their blood pressure was not controlled with antihypertensive agents. Next, 17 patients were lost during follow-up due to relocation and non-compliance throughout the follow-up. Furthermore, we excluded 78 patients with stroke at baseline to rule out the reverse causality. Eventually, 1,107 study subjects with OSA aged ≥ 60 years were included in the longitudinal analysis (Figure 1). Table 3 presents the intergroup comparison of baseline characteristics between the groups with AO and without AO. The incidence of new stroke was 5.9% in approximately 4 years in the prospectively followed-up older patients with OSA. The mild, moderate, and severe OSA groups had overall cumulative incidence rates of stroke of 5.2, 5.4, and 6.7%, respectively. Patients with AO had a higher cumulative incidence of stroke than those without AO (8.3% vs. 2.4%; *P* < 0.05), and participants with severe OSA and concomitant AO were more likely to have experienced stroke (9.8% *vs*. 2.3%, *P* < 0.05; Figure 3) at the median follow-up of 42 months.

The results of the longitudinal analysis are shown in Table 4. Participants with AO were at greater risk of experiencing stroke than those without AO in the entire cohort (*P* < 0.05). After adjusting for age, sex, BMI, neck circumference, drinking status, smoking status, waist–hip ratio, WC, BMI, sleep parameters, and baseline self-reported comorbidities, the differences were moderately attenuated but remained significant (HR 2.16, 95% CI 1.12–4.04). Table 4 shows the results of the risk of stroke resulting from the AO, with and without at baseline, in the multivariate analysis among the three subgroups according to OSA severity. Each 1-mm increase of WC was associated with a 3% (2–5%), 4% (1–7%), 4% (3–6%), and 3% (2–4%) increase in HR for stroke risk in all enrolled older patients with OSA and in the mild, moderate, and severe OSA groups, respectively (*P* < 0.05; Table 4). The crude hazard ratios (HRs) and adjusted HRs of the longitudinal association between AO and stroke in both the unadjusted model and the multivariable-adjusted model were statistically significant in the severe OSA group (*P* < 0.05) but not in the mild and moderate OSA groups (*P* > 0.05).

**TABLE 4:** Cox regression model for stroke according to AO among older patients with OSA at a median follow-up of 42-months (*n* = 1,107).

	Model 1		Model 2		Model 3	
			
	*HR* (95*%CI*)	*P*-value	*HR* (95*%CI*)	*P*-value	*HR* (95*%CI*)	*P*-value
**Total participants**						
HR per 1 mm	1.05 (1.03, 1.06)	<0.001	1.04 (1.03, 1.06)	0.011	1.03 (1.02, 1.05)	0.013
Abdominal obesity	3.48 (1.82, 6.67)	<0.001	3.46 (1.81, 6.64)	0.015	2.16 (1.12, 4.04)	0.026
**Mild OSA**						
HR per 1 mm	1.05 (1.02, 1.09)	0.001	1.05 (1.02, 1.08)	0.001	1.04 (1.01, 1.07)	0.001
Abdominal obesity	2.85 (0.81, 10.06)	0.104	2.01 (0.70, 7.77)	0.113	1.84 (0.43, 6.23)	0.214
**Moderate OSA**						
HR per 1 mm	1.05 (1.03, 1.07)	<0.001	1.04 (1.02, 1.06)	0.002	1.04 (1.03, 1.06)	0.014
Abdominal obesity	2.84 (0.95, 8.51)	0.062	2.24 (0.81, 6.94)	0.073	1.98 (0.73, 6.45)	0.081
**Severe OSA**						
HR per 1 mm	1.04 (1.02, 1.06)	<0.001	1.04 (1.02, 1.05)	0.016	1.03 (1.02, 1.04)	0.022
Abdominal obesity	4.69 (1.63, 13.51)	0.004	4.01 (1.52, 11.43)	0.021	3.67 (1.41, 9.87)	0.031

HR, hazard ratio; CI, confidence interval.

Model 1 was unadjusted.

Model 2 adjusted for age and gender.

Model 3 adjusted for BMI, neck circumference, drinking status, smoking status, waist-hip ratio, waist circumference, BMI, sleep parameters, and baseline self-reported chronic diseases.

### Sensitivity analysis

Considering the AO being defined as a WHR ≥ 0.9 for men and ≥0.85 for women, we present the result of WHR as an AO indicator to illustrate the associations between AO and stroke in Supplementary Tables S2, S3, respectively. These associations were approximately consistent across the result confirmed by using the WC as an AO indicator, whether analyzed as a continuous or a categorical variable. Apart from this, we further compared the results of longitudinal analysis by plotting the ROC curves of different obesity measurement parameters included in this study on the ability to identify stroke risk in older patients with OSA. Our results confirm that waist circumference in the AO measure has a higher predictive ability compared to BMI and WHR for the risk of a median incident stroke within approximately 4 years in older patients with OSA (Supplementary Figure S1). Meanwhile, to determine whether CPAP treatment of older patients with OSA has an impact on the results of the stroke outcome during a median follow-up period of 42 months. We divided the 1,107 older OSA patients into three groups, including the reference group (AHI > 5 event/h; CPAP not used; *n* = 915), the untreated OSA (AHI > 5 event/h; CPAP compliance < 4 h/day; *n* = 121), and the CPAP-treated OSA (AHI > 5 event/h; CPAP compliance ≥ 4 h/day; *n* = 71). A comparative analysis was performed to examine the longitudinal association between AO and stroke among three groups according to the CPAP therapy (Supplementary Table S4). Our study found that patients undergoing CPAP therapy for OSA (CPAP compliance ≥ 4 h/day or CPAP compliance<4 h/day) fell short of statistical significance for the risk of stroke compared to patients with OSA without undergoing the CPAP therapy (Supplementary Table S4).

## Discussion

This is the first study to explore the relationship between AO and stroke among Asian older OSA patients based on a multicentric, observational database. We found that AO was associated with both strokes at the baseline and risk of stroke at the median follow-up of 42 months. Moreover, with AO as a continuous or categorical variable, the association between AO and stroke in older patients varied with the OSA subtype.

The prevalence of AO among older patients with OSA in the present study was 12.4%, which is higher than the prevalence reported in other studies among selected different older general populations (Caspard et al., 2018; Seo et al., 2018; Zhang et al., 2019). Several studies uncovered a specific association between AO and stroke. In one study (Suk et al., 2003) that investigated the relationship between AO and stroke, the main result was that AO is an independent, potent risk factor for stroke among all race-ethnic community-dwelling adults in northern Manhattan, NY, United States. A prospective cohort study among 36,632 adults aged 18–90 was conducted using the China Chronic Disease Risk Factor Surveillance and showed that both normal weight or underweight with AO and overweight or obesity with AO adults had a higher incidence risk of stroke compared to those patients with normal weight, underweight, or obesity without AO during an average follow-up of more than 6 years (Cong et al., 2022). Another study among older participants (mean age 63.0 ± 8.4 years) conducted using the Reasons for Geographic and Racial Differences in Stroke (REGARDS) national cohort demonstrated a significant association between AO and the risk of atrial fibrillation (Kim et al., 2022). Atrial fibrillation is the main risk factor for stroke due to the associated hypercoagulability, leading to arterial embolism (Seiffge et al., 2019). Liu S. et al. (2020) used data from a population-based cohort study comprising more than 26,815 Chinese adults aged ≥ 35 years and found that higher levels of AO consistently predicted an increased risk of stroke during an 11.8-year follow-up.

In contrast, previous studies investigated the relationship directly between general adiposity and stroke but yielded conflicting results. Different methodological approaches, follow-up periods, baseline variables, and study samples might account for discrepancies across studies, as will be discussed later. Abete et al. (2015), based on the data from the 41,020 Spanish EPIC participants aged 26–69 years, confirmed that BMI, an indicator of general adiposity, was not associated with stroke incidence after adjusting for all potential confounders. Moreover, Bodenant et al. (2011) demonstrated that WC, an indicator of AO, is more strongly associated with stroke risk than BMI, which is in concordance with the results of Liu S. et al. (2020). They observed a continuous and positive association between adiposity (regardless of the general or abdominal adiposity) and stroke (Liu S. et al., 2020). However, considering that AO is associated with an increased risk of stroke, whether AO has a stronger association with stroke among patients with OSA remains unclear, especially the elderly population. Our results may add another piece of evidence to partially support the importance of increasing levels of AO for patients with OSA. We selected the older patients with OSA with a median age in the mid-60s, which confirmed the link between AO and the risk of stroke and provides a better direction for preventive interventions.

Notably, regardless of the BMI categories, the association of AO with a high probability of the prevalence and incidence risk of stroke in older patients with OSA were moderately attenuated but remained after controlling for variables, such as age, sex, BMI, neck circumference, drinking status, smoking status, waist–hip ratio, WC, BMI, sleep parameters, and baseline self-reported chronic diseases at baseline. This indicates that the AO association to stroke does not largely change with the mixture of other factors at baseline in patients with OSA. Additionally, the results of this study confirm the prospective association between AO and stroke in patients with OSA. The subgroup analysis further found that the correlation of this risk was mainly reflected in patients with severe OSA, which suggests that the obesity paradox in older may serve as a certain protective effect on the cerebrovascular system for Chinese older patients with mild to moderate OSA during a prospective median follow-up of 42 months. Furthermore, older patients with OSA are known to have good tolerance to chronic intermittent hypoxia and maintenance of cerebral blood flow through brain ischemic preconditioning in the early stages (Sforza and Roche, 2016). The cross-sectional analysis revealed an association between AO and stroke, which was stronger among participants with moderate OSA only. One potential reason may be the moderate patients with OSA with higher age, which attenuates the self-renewal capacity of the body. Catalan-Serra et al. (2019) found a different association between the severity of OSA and stroke. The age did vary across different groups and the different severity of OSA among older patients concomitant with different chronic diseases at baseline will have different compensatory power to continuous intermittent hypoxia, which might explain the relative difference of AO exerted on the incidence of stroke in different OSA groups. Certainly, this is a significant issue that deserves further exploration and discussion in future works.

However, our study does not show that CPAP treatment has a differential influence on the association between AO and stroke outcome in older patients with OSA. This is likely due to the fact that most of the patients regularly treated with CPAP in this study were patients with mild to moderate OSA, who show modest tolerance to recurrent episodes of intermittent hypoxia during sleep, and also the obesity paradox phenomenon may play a moderate protective role in patients with mild to moderate OSA. This issue deserves further exploration in the future.

Although the exact mechanism remains unclear, there are several potential pathways through which AO can impact the stroke incidence rates of HRs and the prevalence rates of odds in older patients with OSA. First, WC was abnormally elevated and, as a measure that is an indicator of AO, is one of the major chronic complications of metabolic syndrome, as well as hyperglycemia and hypertension. Next, hyperglycemia has also been demonstrated to promote the formation of advanced glycation end products, which can stimulate atherogenic endothelial damage (Francisco et al., 2019; Kang et al., 2020). At the same time, previous studies confirmed that chronic intermittent hypoxia-induced adenosine-triphosphate depletion and lactate accumulation were found to promote atherosclerotic due to the thickening of blood vessel walls (Lee et al., 2017; Liu H. et al., 2020). These processes might be associated with the development of stroke. Second, excessive adipose tissue releases various bioactive and inflammatory mediators that affect the body’s metabolism, immune, and thrombolytic pathways that are associated with stroke risk. Most of these factors are overproduced with obesity. Conversely, levels of adiponectin are downregulated during obesity (Tschritter et al., 2003; Lihn et al., 2005; Hajri et al., 2017; Głowinska-Olszewska et al., 2020; Skorepa et al., 2020). Wang et al.’s (2019) results showed that high adiponectin is associated with stroke severity and support the hypothesis that adiponectin can serve as a biomarker of poor outcome after stroke, independent of baseline variables. Thus, the decrease of adiponectin levels caused by AO and the recurrent hypoxia-reoxygenation in patients with OSA may activate oxidative stress, increase sympathetic excitability, and decrease adiponectin levels, both of which might increase the risk of stroke during the follow-up period (Giatti et al., 2021). Third, AO, rather than general obesity, is a major predisposing factor for patients with OSA, especially in the Chinese population (Zhao et al., 2019; Qin et al., 2021). Zhao et al. (2019) revealed a linear dose–response relationship between OSA and AO. In the longitudinal study, the change in WC was significantly correlated with ΔAHI^12^.

The strengths of this study included its prospective design, the inclusion of subjects who received a gold standard for diagnosis of overnight PSG study, and potential confounding factors controlled by both performing cross-sectional analysis and longitudinal analysis to identify the association between AO and the risk of stroke among totally enrolled older patients with OSA as well as in different OSA groups. However, our study has several limitations that should be acknowledged. First, the main limitation of the present study is that the participant is racially and ethnically homogenous, which is a potential limiting factor to the generalization of our results. Second, the absence of analysis of patients’ change dynamically in AO could not account for the longitudinal impact of AO on stroke in this study. Third, some patients did not report the onset of a new- stroke with regard to a relatively short follow-up period of our study, with a median follow-up of just 42 months. Finally, stroke risk of older patients with OSA is mediated by a complex process and is associated with multiple factors that we did not measure (e.g., disease-specific fears); also, other factors that are potentially affected by AO were not evaluated. Accordingly, future studies taking these issues into account are required to validate and expand the current findings. However, we do not consider that these limitations negate the value of our study.

## Conclusion

In this Chinese population-based multicenter study, we provide evidence in support that AO was associated with stroke both at baseline and during a prospective median follow-up of 42 months. Again, considering the specific OSA groups, the relation was varied because of the different categories of variables in AO. Our results showed that the “obesity paradox” cannot be regarded as a major concern in an older population with OSA. Additionally, stroke is associated with increased risks of all-cause mortality and other adverse outcomes; thus, special attention should be paid to the care of Chinese older OSA populations concomitant with AO, and early screening of AO should be scaled up to prevent and reduce the prevalence and incidence of stroke burden.

## Data availability statement

The datasets presented in this article are not readily available for confidentiality reasons. Requests to access the datasets should be directed to the authors, without undue reservation.

## Ethics statement

The Ethics Committee of Chinese PLA General Hospital (S2019-352-01) approved the study. Written informed consent was obtained from all participants.

## Author contributions

YHG, YG, JG, JL, and YY collected and analyzed the data. XS, KL, and LY wrote and participated in all aspects of this research, including the field investigation. LL, JH, and KC designed this study. All authors have read and approved the submitted manuscript.

## Abbreviations

AO: abdominal obesity
OSA: obstructive sleep apnea
PSG: polysomnography
AHI: apnea–hypopnea index
TST: total sleep time
TSA90: duration of time with SaO_2_ < 90%
ODI: oxygen desaturation index
MSpO_2_: mean pulse oxygen saturation
LSpO_2_: lowest pulse oxygen saturation
BMI: body mass index
NC: neck circumference
WC: waist circumference
WHR: waist/hip ratio
CHD: coronary heart disease
COPD: chronic obstructive pulmonary disease
CAS: carotid atherosclerosis
AF: atrial fibrillation.
