# Supplementary material for: Associations between abdominal obesity and the risk of stroke in Chinese older patients with obstructive sleep apnea: Is there an obesity paradox?
Source: Front Aging Neurosci. 2022 Sep 12;14:957396. doi: 10.3389/fnagi.2022.957396 (PMC9510899; doi:10.3389/fnagi.2022.957396)
Supplement: Supplementary file 1 [file Data_Sheet_1.docx]

**Supplementary materials:**

Table s-1 Characterics of covariates

| Covariates | Category | Definition |
| --- | --- | --- |
| Gender, n (%) | Categorical variables | Male, female |
| Age, year | Continuous variable |  |
| BMI, kg/m^2^ | Continuous variable |  |
| Smoking, n (%) | Categorical variables | Never or ever smoking, and current smoking |
| Drinking , n (%) | Categorical variables | Never or ever alcohol drinking, and current alcohol drinking |
| Waist circumference, cm | Continuous variable |  |
| Neck circumference, cm | Continuous variable |  |
| Waist-hip ratio | Continuous variable |  |
| TST, h | Continuous variables |  |
| AHI, events/h | Continuous variable |  |
| ODI, events/h | Continuous variable |  |
| MSpO_2_, % | Continuous variables |  |
| LSpO_2_, % | Continuous variables |  |
| TSA90, min | Continuous variables |  |
| AO, n (%) | Categorical variables | Yes, No |
| CHD, n (%) | Categorical variables | Yes, No |
| Hyperlipidemia, n (%) | Categorical variables | Yes, No |
| Hypertension, n (%) | Categorical variables | Yes, No |
| Atrial fibrillation, n (%) | Categorical variables | Yes, No |
| Carotid atherosclerosis, n (%) | Categorical variables | Yes, No |
| COPD, n (%) | Categorical variables | Yes, No |
| Diabetes, n (%) | Categorical variables | Yes, No |

BMI: body mass index; AHI: the apnea-hypopnea index; ODI: the oxygen desaturation index; MSpO_2_: the mean pulse oxygen saturation; LSpO_2_: the lowest pulse oxygen saturation; TST: total sleep time; TSA90: the duration of time with SaO_2_<90%; AO: abdominal obesity; CHD: coronary heart disease; COPD: chronic obstructive pulmonary disease.

**Table s-2** Logistic regression model for stroke according to AO among older patients with OSA at baseline (n=1290, AO was assessed from the waist-hip ratio)

|  | **Unadjusted** analysis | **adjusted** analysis |
| --- | --- | --- |
|  | **OR (95%CI )** | **OR (95%CI )** |
| **Total participants** |  |  |
| OR per 1mm | 1.02 (1.01, 1.04)** | 1.02 (1.01, 1.03)* |
| abdominal obesity | 1.93 (1.25, 2.91)** | 1.86 (1.18, 2.72)* |
| **Mild OSA** |  |  |
| OR per 1mm | 1.02 (1.01,1.04)** | 1.02 (1.01,1.03)* |
| abdominal obesity | 3.06 (0.99, 9.38) | 2.94(0.86, 9.05) |
| **Moderate OSA** |  |  |
| OR per 1mm | 1.04 (1.01, 1.05)* | 1.03(1.01, 1.04)* |
| abdominal obesity | 2.10 (1.09, 2.98)* | 1.91 (1.04, 2.51)* |
| **Severe OSA** |  |  |
| OR per 1mm | 1.02 (1.01, 1.04)** | 1.02 (1.01, 1.03)* |
| abdominal obesity | 1.14 (0.76, 1.89) | 1.10 (0. 57, 1.63) |

OR, odds ratio; CI, confidence interval. **P* < 0.05. ***P* < 0.01. ****P* < 0.001.

**Table s-3** Cox regression model for stroke according to AO among older pateints with OSA at median 42-month follow-up (n=1107, AO was assessed from the waist-hip ratio)

|  | **Unadjusted** analysis | **adjusted** analysis |
| --- | --- | --- |
|  | ***HR* (95*%CI* )** | ***HR* (95*%CI* )** |
| **Total participants** |  |  |
| OR per 1mm | 1.03 (1.01, 1.04)** | 1.03 (1.02, 1.04)* |
| abdominal obesity | 2.15(1.73, 3.45)** | 1.67 (1.51, 2.81)* |
| **Mild OSA** |  |  |
| OR per 1mm | 1.04 (1.02, 1.06)* | 1.03 (1.02, 1.05)* |
| abdominal obesity | 1.61 (0.73, 2.69) | 1.42 (0.44, 2.13) |
| **Moderate OSA** |  |  |
| OR per 1mm | 1.03 (1.02, 1.06)** | 1.03 (1.02, 1.05)* |
| abdominal obesity | 2.84(0.95, 8.51) | 2.24 (0.81, 6.94) |
| **Severe OSA** |  |  |
| OR per 1mm | 1.04 (1.02, 1.05)** | 1.03 (1.01, 1.05)* |
| abdominal obesity | 3.65 (1.13, 4.47)** | 2.98 (1.07, 3.72)* |

HR, Hazard ratio; CI, confidence interval. **P* < 0.05. ***P* < 0.01. ****P* < 0.001.

**Table s-4.** Subgroup analysis of the longitudinal associations between AO diabetes and stroke

|  | **Unadjusted analysis** | | **Adjusted analysis** | |
| --- | --- | --- | --- | --- |
|  | **HR (95%CI )** | ***P*-Value** | **HR (95%CI )** | ***P*-Value** |
| Reference group | 1.00 (ref.) |  | 1.00 (ref.) |  |
| Untreated OSA group | 0.22 (0.03, 1.56) | 0.129 | 0.21 (0.03, 1.52) | 0.123 |
| CPAP-treated OSA group | 0.82 (0.33, 2.04) | 0.661 | 0.67 (0.26, 1.68) | 0.391 |

Reference group (AHI＞5 event/hours, CPAP not used); Untreated OSA group (AHI＞5 event/hours；CPAP compliance＜4hours/day); CPAP-treated OSA group (AHI＞5 event/hours；CPAP compliance≥4hours/day)


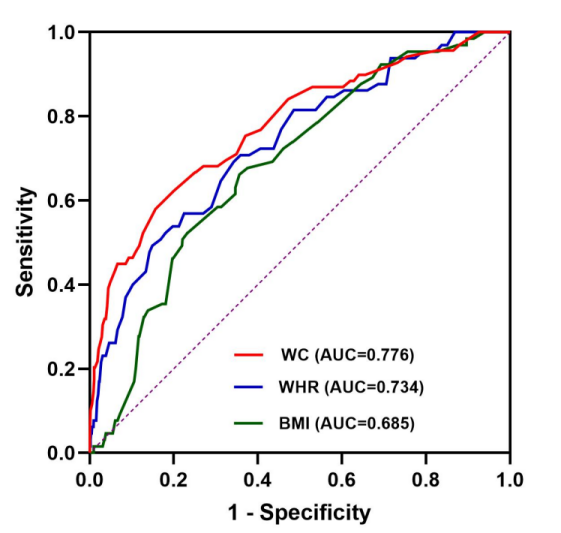


**Figure s-1.** The capability of different obesity measurement parameters to identify the patients of OSA with a risk of stroke. Receiver-operating characteristic curve showed the performance of WC, WHR, and BMI in predicting stroke outcome, respectively. The AUC was 0.776 (95% CI: 0.714– 0.838, *P*＜0.05), 0.734 (95% CI: 0.670– 0.798, *P*＜0.05), and 0.685 (95% CI: 0.624– 0.745, *P*＜0.05). AUC: area under the receiver operating characteristic curve; CI: confifidence interval; BMI: body mass index; NC: neck circumference; WC: waist circumference;WHR: waist/hip ratio.
